# Age-Dependent Decline of Endogenous Pain Control: Exploring the Effect of Expectation and Depression

**DOI:** 10.1371/journal.pone.0075629

**Published:** 2013-09-27

**Authors:** Wiebke Grashorn, Christian Sprenger, Katarina Forkmann, Nathalie Wrobel, Ulrike Bingel

**Affiliations:** 1 Department of Neurology, University Medical Center Hamburg-Eppendorf (UKE), Hamburg, Germany; 2 Department of Systems Neuroscience, University Medical Center Hamburg-Eppendorf (UKE), Hamburg, Germany; University of Arizona, United States of America

## Abstract

Although chronic pain affects all age ranges, it is particularly common in the elderly. One potential explanation for the high prevalence of chronic pain in the older population is impaired functioning of the descending pain inhibitory system which can be studied in humans using conditioned pain modulation (CPM) paradigms. In this study we investigated (i) the influence of age on CPM and (ii) the role of expectations, depression and gender as potential modulating variables of an age-related change in CPM. 64 healthy volunteers of three different age groups (young = 20–40 years, middle-aged = 41–60 years, old = 61–80 years) were studied using a classical CPM paradigm that combined moderate heat pain stimuli to the right forearm as test stimuli (TS) and immersion of the contralateral foot into ice water as the conditioning stimulus (CS). The CPM response showed an age-dependent decline with strong CPM responses in young adults but no significant CPM responses in middle-aged and older adults. These age-related changes in CPM responses could not be explained by expectations of pain relief or depression. Furthermore, changes in CPM responses did not differ between men and women. Our results strongly support the notion of a genuine deterioration of descending pain inhibitory mechanisms with age.

## Introduction

Chronic pain represents one of the largest medical health problems in the developed world and affects about 19% of the adult population [1]. Although chronic pain affects all age groups, it is particularly common in the elderly population affecting more than 50% of the older adults and up to 80% of nursing home residents [2]. Yet there are surprisingly few studies addressing pain in the elderly and its underpinning pathophysiology [3]. Contemporary models of pain emphasize that the susceptibility to acute and chronic pain states is determined by the balance of ascending and descending pain modulatory pathways [4], [5]. Accordingly, the high prevalence of pain among the elderly might, at least in part, be explained by age-dependent changes in the descending pain modulatory system.

The descending pain modulatory system can selectively modulate pain by either inhibiting or facilitating nociceptive processing [4]. A well-established tool to study the descending pain control system in humans is the use of conditioned pain modulation (CPM) paradigms. In these paradigms pain intensity ratings of test stimuli ( = TS, e.g. heat pain stimuli applied to the arm) are obtained with and without concomitant application of a second pain stimulus (i.e., the conditioning stimulus = CS) that is applied to another part of the body (e.g. a cold pressor task applied to the leg). Positive CPM responses, which are defined by a lower pain intensity rating for the test stimuli when they are applied in combination with the conditioning stimulus, are indicative of endogenous analgesia. The corresponding phenomenon in animals termed DNIC (diffuse noxious inhibitory controls) is known to be based on a basal spino-bulbo-spinal reflex [6], [7]. In contrast, CPM responses in humans have been shown to underlie cognitive manipulations [8] and to involve higher cortical brain areas such as the cingulate and frontal cortex [9]–[11].

Given the high prevalence of pain among the elderly, surprisingly few studies have investigated age-dependent changes in endogenous pain control mechanisms so far. Previous CPM studies involving older participants support the notion that the capacity of endogenous pain control may decrease with age [12]–[16]. In many of these studies only pain thresholds and not pain ratings of suprathreshold stimuli had been obtained. This, however, might be more appropriate to assess changes in clinical pain conditions. Furthermore, most of the studies did not explore the influence of cognitive-emotional factors known to impact pain modulatory mechanisms and to change with age. In this study, we investigated (i) whether CPM responses decrease with age and (ii) whether the relationship between CPM responses and age is modulated by cognitive and emotional factors. To this end we used a well-established CPM paradigm [11] in three age groups, namely young, middle-aged and elderly healthy subjects. To assess the potential influence of cognitive-emotional factors, we studied the effects of expectation and depression on CPM responses as well as the influence of gender which have been previously discussed as potentially modulating variables [17], [18].

## Methods

### 1. Participants

78 potential participants of three different age groups (20–40 years, 41–60 years, 61–80 years) were recruited locally and enrolled in the study if they fulfilled the following inclusion criteria: (1) between the age of 18 and 80 years, (2) free from chronic pain, (3) not currently using any prescription analgesics, tranquilizers, antidepressants, anticonvulsants, (4) not pregnant. Of 78 potential participants, 14 were excluded from the final data analysis for the following reasons: 11 subjects did not finish the protocol (they withdrew from the experiment during the cold pressor task) and 3 subjects did not meet the inclusion criteria at the day of the experiment (HADS Anxiety ≥11). Thus data analyses were based on 64 complete data sets: 22 young adults (mean age 24.8±2.8, 12 female, 10 male), 17 middle-aged adults (48.7±5.3, 9 female, 8 male) and 25 older adults (70.3±5.2, 13 female, 12 male). The study was conducted in accordance with the Declaration of Helsinki and was approved by the local Ethics Committee of the Medical Council of Hamburg. All participants gave written informed consent and were free to withdraw from the study at any time.

### 2. Experimental protocol

**Figure 1:**
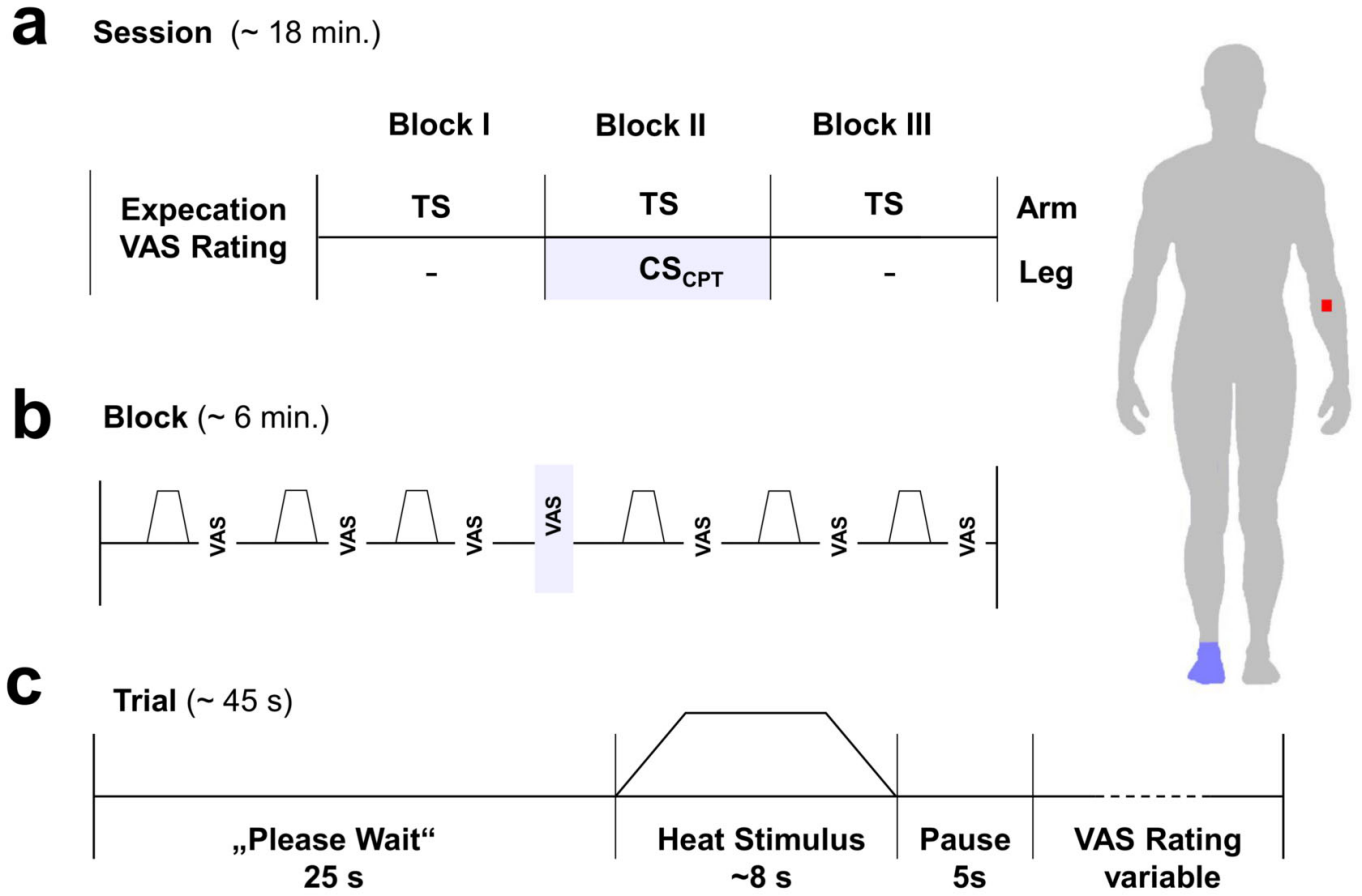
Experimental design. The experiment consisted of three blocks á six painful heat stimuli ( = test stimuli) applied to the right forearm. During block one and three only the test stimuli were applied, whereas in block two a concomitant cold pressor task to the contralateral foot was applied as conditioning stimulus. Pain ratings were obtained of each test stimulus and once of the conditioning stimulus in the middle of block two. Before the actual experiment started, the a priori expectation of each participant regarding pain intensities of test stimuli during the cold pressor task was assessed.

In this study we used a well-established CPM paradigm [11] which combines painful heat stimuli as test stimuli (TS) with a cold pressor task as the conditioning stimulus (CS). In brief, the experimental procedures included an introductory session which consisted of filling in several questionnaires and the calibration of stimulus intensities. This was followed by the a priori assessment of expectation regarding possible changes of pain intensities during the application of the cold pressor task. Finally, the actual CPM paradigm was performed, that consisted of three blocks, in which six test stimuli each were applied. Pain ratings to these stimuli were obtained before ( = block I), during ( = block II) and after ( = block III) a cold pressor task that was applied to the leg during the second block. The experimental protocol is summarized in figure 1 and described in detail below.

#### 2.1. Instructions and calibration procedure

All participants were instructed using a standardized protocol. Participants were told that the purpose of the study was to characterize possible differences in the perception of two simultaneously applied painful stimuli comparing healthy participants of different age groups (young, middle-aged and older adults). First, participants were informed about the sequence of experimental procedures. After these general instructions, subjects filled in the HADS questionnaire and an assessment of acute pain was performed. If they fulfilled all inclusion criteria (see 2.1) a calibration procedure was performed to determine the individual temperatures corresponding to a pain level of 50–60 on a 0–100 visual analogue scale [VAS, endpoints 0–100]. To this end, we pseudo-randomly applied six stimuli á six seconds each with different intensities ranging from 45.5–49.5°C to the right volar forearm, every temperature was presented once. Participants were asked to rate the intensity of each stimulus on a VAS which was presented on a computer screen in front of the subjects and ranged from 0 = “no sensation” to 100 = “most intense pain imaginable”. Two vertical white lines represented the two endpoints 0 and 100 of the VAS, a third white line was set at 25 labeled as “pain threshold”. Subjects indicated the pain intensity of each heat pain stimulus by moving a red bar between the two endpoints using two buttons of a computer mouse. The maximum stimulation temperature was restricted to 49.5°C in order to avoid any tissue damage. This calibration procedure ensured that all participants perceived the phasic heat pain stimuli ( = test stimuli, TS) as equally painful (VAS 50–60).

The application of the thermal stimuli, the presentation of the VAS and the recording of behavioral data was performed using the software Presentation (www.neurobs.com).

#### 2.2. Test stimulus

We used phasic heat pain stimuli as test stimuli (TS). The test stimuli were applied to the right volar forearm (∼10 cm proximally from the wrist) of the participants using a 30×30 mm Peltier-Thermode (TSAII, Medoc, Israel). Each stimulus had a duration of six seconds (baseline temperature 35°C, ramp up and down 10°C/second, destination temperature individually calibrated between 45.5 and 49.5°C, interstimulus-interval ∼45 seconds). Pain ratings on the VAS were obtained immediately after each stimulus. A total of 18 test stimuli were applied. The first ( = *block I*, stimulus one to six) and the last six stimuli ( = *block III*, stimulus 13–18) were applied without any other concomitant procedures. During the application of test stimuli seven to twelve ( = *block II*), the conditioning stimulus was applied.

#### 2.3. Conditioning stimulus

A cold pressor task was used as the conditioning stimulus (CS). After completion of the first block of six heat pain stimuli (*block I*), a message on the computer screen prompted the participants to immerse their left foot into a bath with ice water (∼0°C). The intensity of the conditioning stimulus was rated once in the middle of the cold pressor task ( = after TS 9, block II) using a VAS presented on a computer screen with the same endpoint labels 0 = “no sensation” and 100 = “most intense pain imaginable” and a third white line set at 25 labeled as “pain threshold”. At the end of block II another message on the computer screen instructed the participants to take their foot out of the ice water. Prior to the experiment subjects were asked to focus their attention on the heat stimuli applied to the arm while having their foot immersed into the ice water and it was pointed out again that they could withdraw from the experiment at any time by telling the supervising experimenter. Finally heat pain stimuli 13–18 (block III) were applied without concomitant painful stimulation to the foot.

#### 2.4. Assessment of individual expectation

Following the calibration procedure, immediately prior to the actual experiment, patients were presented the following question on the computer screen: “How do you expect the pain applied to your arm to change while you have your foot immersed into the ice water?” Participants marked their expectations on a VAS with the verbal anchors 0 = “no sensation” ( = meaning that the pain at the arm would be completely abolished during the cold pressor task), 50 = “no change” ( = no change of heat pain at the arm during the cold pressor task), and 100 = “maximum pain” ( = pain applied to the arm would get worse during the cold pressor task and when marking 100 the experiment would not be continued).

#### 2.5. Assessment of anxiety and depression

The Hospital Anxiety and Depression Scale (HADS) [19] is a self-report questionnaire to assess anxiety and depression with 7 items per subscale. Each item is scored from 0–3 points so that scores of 21 points for each subscale depression and anxiety can be reached, higher scores indicating higher symptom severity. Both subscales have been validated to have good sensitivity and specificity [20].

### 3. Data analysis

As in previous studies [21], [22] the CPM response was calculated as the difference between mean pain ratings before and after the cold pressor task and mean pain ratings during the cold pressor task (CPM response = mean pain ratings block (I+III) - blockII). A positive CPM response indicates a reduction in pain perception during the cold pressor task and therefore signifies analgesia symbolizing effective descending pain inhibition mechanisms, whereas a negative CPM response shows an increase of pain ratings in block II. We also calculated differences between mean pain ratings of block I and block II (block I–II) to look specifically at the differences in pain ratings only before and during the cold pressor task. All data analyses were carried out using SPSS 18.0 and Matlab.

To test if there was a significant CPM response in each of the three different age groups, we conducted three separate one sample t-tests for every single age group. Group differences of CPM responses between the three age groups were analyzed using non-parametric Kruskal-Wallis tests as variables in the different age groups were not normally distributed. Significant main effects were followed by post-hoc Mann-Whitney U tests.

One way analysis of variance (ANOVA) or the appropriate non-parametric tests (Kruskal-Wallis) for non-normally distributed variables were used to test for potential differences in pain intensity ratings of the test (block I) and conditioning stimuli (cold pressor task) across groups. The effect of age on CPM response was assessed using a linear regression model with CPM response as dependent variable and age as independent variable. To test for further modulatory influences on the CPM response, we used stepwise multiple regression analysis with CPM response as dependent variable and age, expectation, depression and gender as independent variables ( = predictors). Relationships between the different variables were explored using Pearson product moment correlations and hierarchical regression analyses. *P*<0.05 (two-tailed) was considered statistically significant. Post-hoc Mann-Whitney U tests and t-tests for applied stimulation temperatures, depression scores and CPM responses were conducted using Bonferroni adjusted alpha levels of 0.017 per test (0.05/3) accounting for every independent test within in each age group.

## Results

### 1. Pain intensity ratings of test stimuli in block I, conditioning stimuli, applied stimulation temperatures and expectation

Prior to the hypothesis-driven analyses of age-dependent differences of the CPM response we tested for potential differences in mean pain ratings of test stimuli in block I, of the conditioning stimulus and of the applied stimulation temperatures between the three different groups which could account for possible differences in CPM responses between the three groups. There were no group differences in mean pain ratings of test stimuli in block I (F(2,61) = 1.559, p = 0.219) or in pain ratings for the conditioning stimuli (F(2,61) = 0.327, p = 0.722). In line with the known age-dependent increase in pain thresholds [23], higher stimulation temperatures were needed to elicit pain intensity ratings of 50–60 in the older age groups: there were significant differences of the applied stimulation temperatures between the three age groups (H(2) = 21.5, p<0.001).). Post hoc Mann-Whitney U tests revealed significant differences between young and middle-aged (U = 77.5, z = −2.97, p = 0.003) and young and older adults (U = 51, z = −4.311, p<0.001), whereas no differences between middle-aged and older adults could be detected (U = 102, z = −1.9, p = 0.06).

Also, expectation regarding the effect of the conditioning stimulus on the test stimuli did not differ between the different age groups (H(2) = 3.3 p = 0.188).

Depression scores differed significantly between the three age groups (H(2) = 12.7, p = 0.002). Post hoc Mann-Whitney U Tests showed significant lower depression scores for young participants compared to middle-aged (U = 95.0, z = −2.8, p = 0.005) and older adults (U = 129.0, z = −3.3, p = 0.001) whereas no differences between middle-aged and older adults were found (U = 184.5, z = −0.7, p = 0.464).

Table 1 summarizes descriptive data of psychophysical measures of young, middle-aged and elderly participants.

**Table 1:** Descriptive data of psychophysical measures of young, middle-aged and older participants.

Variable	young	middle-aged participants	older
Test stimuli block I (VAS ratings, mean±SD)	61.2±12.9	58.4±15.5	54.9±8.7
Conditioning stimulus (VAS ratings, mean±SD)	70.1±18.0	75.4±21.9	72.8±21.4
Stimulation temperature (°C, mean±SD)	46.8±0.8	47.7±0.7	48.2±0.8
Expectation (VAS ratings, mean±SD)	−8.3±18.3	−12.6±15.0	−3.9±19.6
HADS_depression score (mean±SD)	0.4±0.6	1.6±1.5	2.2±2.0

### 2. Age and CPM

**Figure 2:**
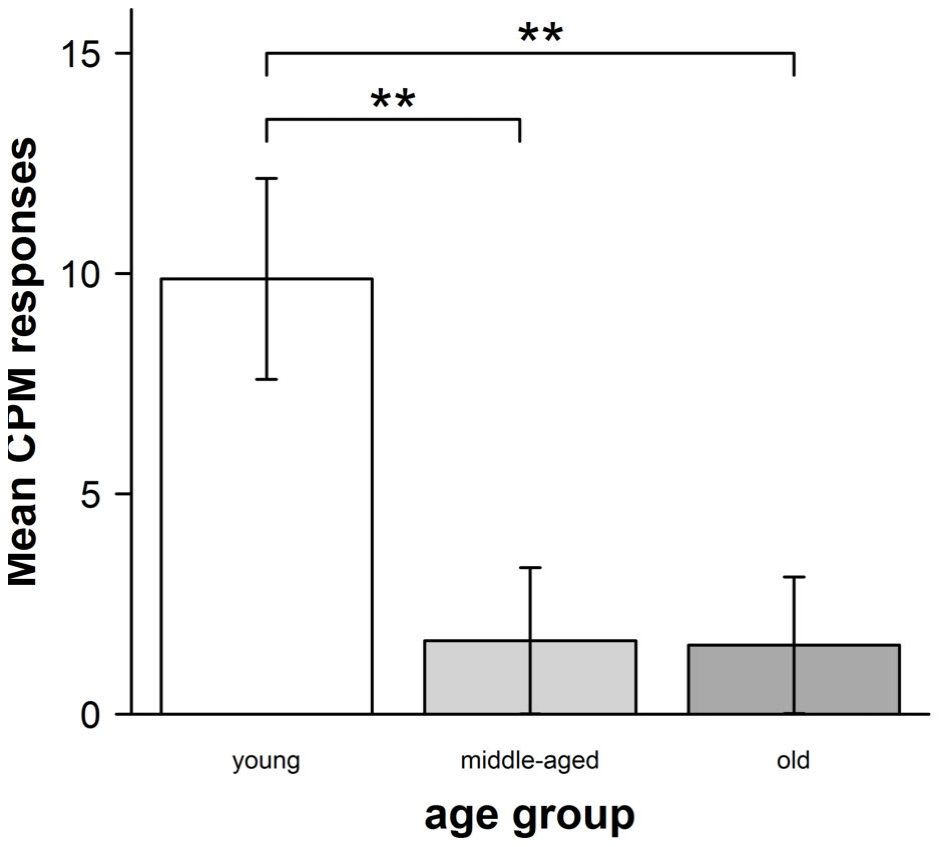
CPM responses in the three different age groups (±SEM). CPM responses were calculated as mean pain intensity ratings of (block 1+3) – block 2. Thus positive CPM responses were indicative of endogenous analgesia.

As hypothesized CPM responses differed significantly between the three age groups (H(2) = 11.4, p = 0.003, see figure 2).

Post-hoc Mann-Whitney U tests revealed significant differences between young and middle-aged adults (U = 102, z = −2.4, p = 0.016) and between young and older adults (U = 122, z = −3.3, p = 0.001), whereas no group differences between middle-aged and older adults could be detected (U = 210.5,z = −0.1, p = 0.959).

Looking at the group-specific CPM responses in more detail, we found that young adults showed a highly significant CPM response (mean CPM response = 9.9, SD = 10.7, t(21) = 4.3, p<0.001, one-sample t-test), whereas middle-aged (mean CPM response = 1.7, SD = 7.7, p = 0.356, Wilcoxon-test) and older adults (mean CPM response = 1.6, SD = 7.1, t(24) = 0.8, p = 0.411, one-sample t-test) did not show significant CPM responses.

**Figure 3:**
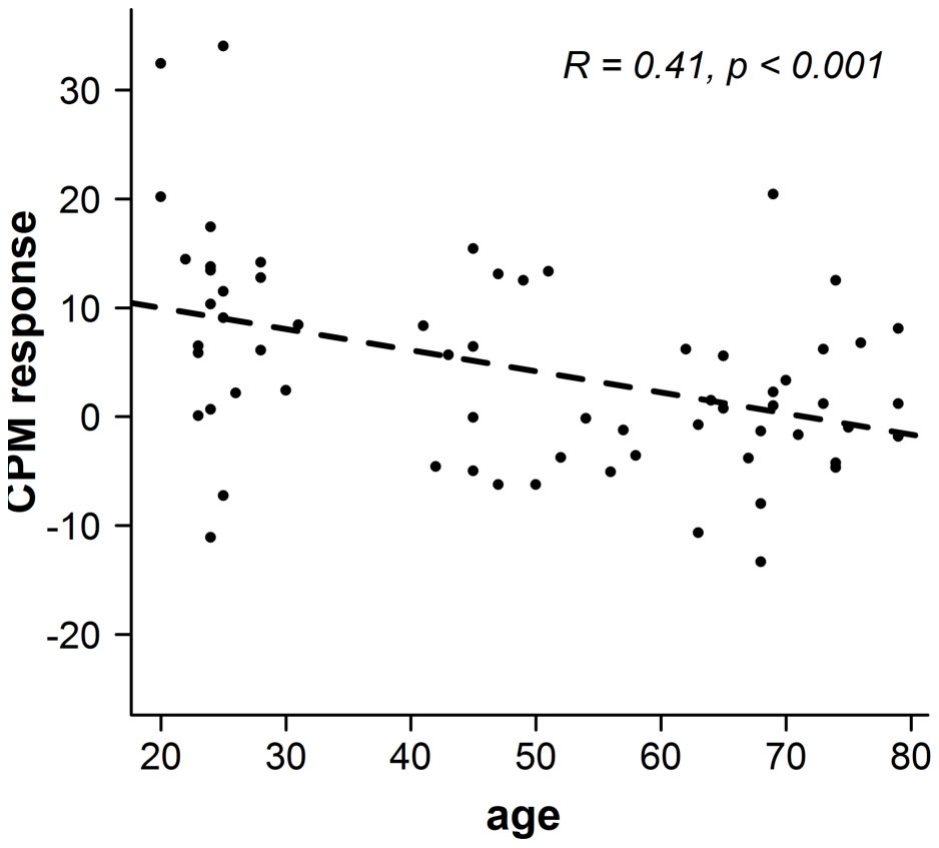
CPM responses and age. CPM responses showed a significant age-dependent reduction with ongoing age as revealed by a linear regression.

Additionally we tested whether the decline in CPM response could be predicted from age using a linear regression analysis. This analysis revealed a significant age-dependent reduction of CPM responses: β = −0.406, t(61) = 4.796, p<0.001. Thus, age explained a significant proportion of variance in CPM responses, R2 = 0.165, F(1,62) = 12.231, p = 0.001 (see figure 3).

When using another strategy of calculating the CPM response, i.e. comparing the mean pain ratings of block I and block II, very similar results were observed: Here again CPM responses differed significantly between the three age groups (F(2,61) = 8.706, p<0.001). Post hoc t-tests showed significant differences between young and middle-aged adults (t(37) = 3.823, p<0.001) and between young and older adults (t(45) = 3.198, p = 0.003), whereas no differences between middle-aged and older adults could be detected (t(40) = −0.743, p = 0.462).

Moreover, young adults showed a highly significant CPM response (mean CPM response = 12.31, SD = 12.66, t(21) = 4.56, p<0.001, one-sample t-test) whereas middle-aged (mean CPM response = −1.61, SD = 9.14, t(16) = −0.726, p = 0.48) and older adults (mean CPM response 0.91, SD 11.77, t(24) = 0.39, p = 0.70) did not.

### 3. Expectation, gender, depression and CPM responses

To identify potential modulating factors regarding the CPM response in more detail, we conducted correlation analyses for CPM response and expectation, gender and depression. We solely found a significant relationship between CPM responses and depression (r = −0.3, p = 0.005). However, the stepwise multiple regression analysis (predictors tested: age and depression) revealed that depression ( = HADS depression score) had no additional influence on CPM responses over and above the influence of age. This might be due to the significant positive relationship between age and depression (r = 0.4, p = 0.001).

The other correlations did not reach statistical significance (CPM and expectation r = −0.05, p = 0.751, CPM and gender χ2 = 60, p = 0.439) and were therefore not included in the above mentioned multiple regression analysis.

## Discussion

In this study we investigated the influence of age on CPM responses and the role of potentially modulating variables, including expectation, depression and gender. Using a well-established CPM-paradigm in three age groups of healthy participants we showed a significant decrease of CPM responses on suprathreshold thermal pain stimuli with age. Importantly, this age-dependent decline in endogenous pain modulatory capacity could not be explained by other factors such as expectation, depression or gender.

### CPM responses and increasing age

Our results of an age-dependent decline in CPM responses confirm previous observations showing an age-dependent reduction of CPM responses [12]–[16]. Washington et al. provided first evidence that the inhibitory effect of a cold pressor task on electrical and thermal laser pain thresholds obtained directly after the task is reduced in an elderly group (mean age 77.9 years) compared to younger participants (mean age 23.3 years) [16]. Edwards et al. reported similar results comparing CPM responses in younger (mean age 21.6 years) and older (mean age 63.1 years) adults using suprathreshold test stimuli. These two studies which provided data on rather extreme age groups did, however, not address the development of deficits in CPM responses with increasing age. A study by Larivière et al. suggested that the decrease of CPM responses already affects middle-aged adults ( = 40–55 years), as not only older (60–75 years) but also middle-aged adults showed decreased CPM responses compared with younger adults (20–35 years) [13]. In this study, however, the inhibitory effect of the conditioning stimulus (cold pressor task) was only assessed on heat pain thresholds, not on suprathreshold stimuli. In a study by Riley et al. [14] older adults (mean age 65.2) were compared with younger adults (mean age 25.3) regarding their CPM responses using a CPM paradigm similar to ours that combined painful heat stimuli to the left palm as test stimuli with or without a concomitant cold pressor task as conditioning stimulus to the contralateral foot. However, they did not assess cognitive-emotional factors like expectation or depression. As in our study, they also found diminished CPM responsesin older adults. Considering the relevance of CPM paradigms particularly for clinical pain syndromes, testing the effect of the conditioning stimulus on suprathreshold stimuli as well as assessing the influence of cognitive-emotional factors like expectation and depression seems warranted. Our study, in which we combined repetitive suprathreshold thermal pain stimuli as test stimuli with a cold pressor task confirms these previous indications of an age-dependent decline of CPM responses. In line with the study by Larivière et al. [13], we show that the decline of CPM responses already affects middle-aged participants. A regression analysis (see figure 2) further suggests a monotone decline with increasing age, rather than an abrupt change at a particular age.

Previous studies have used CPM responses as a valuable marker of endogenous pain modulatory capacity. For instance, variations in CPM responses have been shown to predict the risk for developing chronic pain after certain operations [24]. Accordingly, the age-dependent reduction in CPM responses observed in our study supports the notion that the increasing prevalence for chronic pain disorders with age, is indeed, at least in part, associated with the decline of endogenous pain modulatory control.

### The role of expectation and other modulating variables

The DNIC response that has been characterized in animals represents a basal physiological response that occurs even without the contribution of cortical brain areas [6], [7]. However, the integration of basal reflexes into the control of cortical areas subserving more complex behaviours or cognitive functions is a key feature of evolutionary development. The ability of cognitive factors to influence DNIC or CPM is therefore not only an important but so far understudied topic. As probably best illustrated by models of placebo analgesia [25], expectation is a key factor known to modulate the perception of pain. Yet the literature regarding the role of expectation in CPM responses is sparse. Previous studies have shown that the effects of expectation can interfere with CPM responses. Verbally induced expectations of analgesia or hyperalgesia enhanced or reduced CPM responses in young healthy adults [26], [27]. In a study by Nir et al. it was shown that cognitive manipulation of the perceived pain intensity of the conditioning stimulus resulted in decreased CPM responses: A placebo instruction ( = conditioning stimulus less painful) led to a decreased pain intensity of the conditioning stimulus and a decreased CPM response, whereas a nocebo instruction only resulted in an increased pain intensity of the conditioning stimulus without changes in the CPM response.

However, these observations do not allow for conclusions regarding the role of implicit, not manipulated expectations the participants or patients may hold regarding the CPM procedure. To address this issue, we obtained individual expectancy ratings of how the cold pressor task may influence the perception of the test stimulus prior to the CPM procedure. Our findings show that the expectation does not significantly influence CPM responses and that CPM-related expectations do not change with age. These findings are at least partly in line with a previous study by Larivière et al. who also concluded from their data that the age-dependent reduction of CPM responses cannot be explained by changes in expectation [13].

A second factor that is known to substantially modulate pain perception is depression [28]. Numerous studies have shown a high comorbidity between depression and chronic pain [29]. Furthermore, the prevalence of depression seems to vary across the lifespan with a first peak between 30 and 40 years and a second peak between 50 and 60 years [30]. In this study we obtained scores of the Hospital Anxiety and Depression Score (HADS) from each participant prior to the experiment and correlated depression scores with CPM responses. We found a moderately negative correlation between depression and the magnitude of CPM responses, indicating that subjects scoring higher on the depression subscale showed a reduced CPM response. In order to assess the influence of depression on CPM responses over and above the influence of age we conducted a stepwise multiple regression analysis. Age predicted CPM responses whereas depression did not explain additional variance in CPM responses, and was thus no significant predictor of CPM responses, most likely due to the close relationship between age and depression observed in our data.

In addition to cognitive-affective factors, CPM responses might be gender-sensitive. Numerous studies have shown that the prevalence of chronic pain is higher in women than in men [31]. To date, several studies using different CPM paradigms have addressed gender differences and yielded inconsistent findings. The majority of studies described higher CPM responses in males [17], [32], [33], very few studies reported higher CPM responses in females [34] and others did not show any sex differences [23], [35]–[37] which is in line with the non-significant gender difference in CPM responses across and between all age groups found in the present study.

### Possible underlying mechanisms of age-related changes in CPM responses

The underlying mechanisms in the generation of CPM responses in humans include spinal [6] and supraspinal mechanisms [38], [39] as well as the involvement of higher cortical areas such as the anterior cingulate cortex or prefrontal areas [9], [11].

There is ample evidence for a decrease in brain volume with age [40]–[42]. However, the extent of these changes in brain volume that involves neuronal loss, shrinking of neurons and importantly loss of synapses [43] differs significantly between different brain regions. While large-scale age-related changes in the cerebral cortex are observed, the brainstem shows no relevant [44] or only small volume age-related changes [45]. Yet these small changes in the brainstem could still be responsible for at least parts of the age-dependent decrease of the CPM response. Although we cannot support this notion with brain imaging data, it also appears conceivable that age-dependent morphological changes in higher cortical areas [45] might result in an impaired top-down modulation of CPM responses and hence be a possible explanation for the phenomenon of an age-dependent decrease of CPM responses.

Another important factor might be the reduction in neurotransmitter availability in the elderly. The literature supports a role for both opoidergic [46], [47] and serotonergic [48] pathways for CPM responses. In support of the relevance of serotonergic mechanisms, Yarnitsky et al. recently showed that low CPM responses in patients with painful neuropathy may predict the benefit from the combined serotonine-noradrenaline-reuptake inhibitor duloxetine [49]. Intriguingly, serotonine receptor binding potential declines with age [50] and might thereby contribute to the age-dependent decrease in CPM responses.

### Conclusion

In summary, we show that CPM responses which are indicative of endogenous pain modulation, decline with increasing age. This effect could not be explained by expectation, depression or gender. Our data therefore provide further evidence for a genuine age-dependent change of the descending pain modulatory system. In combination with insights into altered neurotransmitter availability in the elderly, our data could allow for a better understanding of neurophysiological mechanisms underlying pain in the elderly and help the development of improved treatment options for the growing number of older individuals in our population to counteract its detrimental consequences.
